# Experiencing antiretroviral adherence: helping healthcare staff better understand adherence to paediatric antiretrovirals

**DOI:** 10.1186/1758-2652-13-48

**Published:** 2010-12-06

**Authors:** Benjamin R Phelps, Sarah J Hathcock, Jennifer Werdenberg, Gordon E Schutze

**Affiliations:** 1 Baylor College of Medicine, 6621 Fannin, Suite A-150, MS 1-3420, Houston, Texas, USA 77030-2399; 2 Tulane University School of Medicine, 1430 Tulane Avenue, New Orleans, LA 7011, USA

## Abstract

**Background:**

Lack of adherence to antiretroviral medications is one of the key challenges for paediatric HIV care and treatment programmes. There are few hands-on opportunities for healthcare workers to gain awareness of the psychosocial and logistic challenges that caregivers face when administering daily antiretroviral therapy to children. This article describes an educational activity that allows healthcare workers to simulate this caregiver role.

**Methods:**

Paediatric formulations of several antiretroviral medications were dispensed to a convenience sample of staff at the Baylor College of Medicine-Bristol-Myers Squibb Children's Clinical Center of Excellence in Mbabane, Swaziland. The amounts of the medications remaining were collected and measured one week later. Adherence rates were calculated. Following the exercise, a brief questionnaire was administered to all staff participants.

**Results:**

The 27 clinic staff involved in the exercise had varying and low adherence rates over the week during which the exercise was conducted. Leading perceived barriers to adherence included: "family friends don't help me remember/tell me I shouldn't take it" and "forgot". Participants reported that the exercise was useful as it allowed them to better address the challenges faced by paediatric patients and caregivers.

**Conclusions:**

Promoting good adherence practices among caregivers of children on antiretrovirals is challenging but essential in the treatment of paediatric HIV. Participants in this exercise achieved poor adherence rates, but identified with many of the barriers commonly reported by caregivers. Simulations such as this have the potential to promote awareness of paediatric ARV adherence issues among healthcare staff and ultimately improve adherence support and patient outcomes.

## Background

Lack of adherence to antiretroviral (ARV) medications is one of the key challenges for HIV care and treatment programmes [1-3]. While strict adherence promotes viral suppression, poor adherence results in further immunosuppression and resistance to antiretroviral medications [4,5]. Adherence is especially challenging among young infants and children, and supervising daily child dosing requires organizational skills, age-appropriate negotiation skills, and an understanding of how to actually draw up and administer medication to a potentially uncooperative child. Handling paediatric medication can also be a challenge, especially if dispensed as a liquid formulation [6-9].

Paediatric HIV care and treatment remains a global health priority, but health professionals providing ARVs to children are often unaware of these complexities. There are few training initiatives designed to ensure that healthcare providers understand the psychosocial and logistic challenges of taking ARVs on a daily basis. Such an understanding of common barriers is potentially beneficial to effectively discussing adherence strategies with patients and their caretakers [10-12]. Such adherence training exercises also promise to generate ideas and discussion that will lead to improvements in clinical practice and related adherence promotion strategies, both for HIV treatment programmes and those addressing other childhood diseases.

## Methods

Paediatric formulations of highly active antiretroviral therapy (HAART) medications were dispensed on a voluntary basis to a convenience sample of full-time Swazi and expatriate clinical staff at the Baylor College of Medicine-Bristol-Myers Squibb Children's Clinical Center of Excellence in Mbabane, Swaziland. Most clinical staff working on site participated in the exercise, and all who participated were involved in the direct provision of paediatric HIV care and treatment services. Other clinical and non-clinical staff volunteered to offer adherence support to participants in keeping with the adherence support protocol of the clinic. Liquid stavudine, lamivudine and nevirapine were dispensed as per Swaziland care and treatment guidelines (2006 edition). To avoid waste, medications that were used were combined from the small volumes of leftover liquids turned in by patients.

All participating staff attended a group adherence session similar to that offered to caregivers of paediatric clients initiating HAART, including a review of the importance of adherence, the individual ARVs in the regimen and potential adverse effects, the components of successful adherence, and the consequences of poor adherence. Participating staff then read and signed a standard adherence contract and received their assigned three-drug regimen, along with an explanation and dosing calendar from our pharmacists, as per clinic protocol. Participants received liquid formulations of various first-line ARVs as if each was the parent of a young child receiving either an initial ARV regimen or a refill of an ongoing regimen.

During the exercise, participants were asked to adhere strictly to the appropriate schedule, carefully draw appropriate doses, and administer the liquid into the sink, and thus no medications were actually consumed. Participants were requested to keep notes about the experience and the challenges faced and to return with any remaining medications seven days later. At that time, each participant's remaining doses were collected and measured and adherence rates were calculated. Staff adherence was calculated based on overall adherence, which assigned an adherence rate equal to the value of the farthest outlier of the three assigned medications, the same method used routinely in the clinic to calculate client adherence rates.

Each participant also completed a one-page questionnaire derived from the AIDS Clinical Trials Group Self Report survey, which allowed self-reporting on adherence rates and barriers. Each barrier included was weighted using a numeric scale (Zero - "Never a problem"; 1 - "Hardly ever a problem"; 2 - "Frequent problem"; and 3 - "Almost always a problem"). Twelve potential barriers were included in the questionnaire (Table 1). Variables involving cost and side effects were not included in the analysis as all ARVs at the Baylor College of Medicine-Bristol-Myers Squibb Children's Clinical Center of Excellence are provided free of charge, and as participants were disposing of the liquid medicines after drawing them into a syringe, rather than actually administering or consuming the medications.

**Table 1:** Reported barriers to treatment with anti-retroviral therapy at the Baylor-Swaziland paediatric clinic, from most problematic to least problematic (n = 24)

		"Never a problem"	"Hardly ever a problem"	"Frequent problem"	"Almost always a problem"
1	"Got in the way of daily schedule; too busy"	9	5	8	2

2	"Family and/or friends don't help me remember"	7	6	8	3

3	"Difficulty drawing medicine; spillage"	9	9	4	2

4	"Forgot to take medications"	8	9	6	1

5	"Away from home"	9	8	7	0

6	"Didn't feel like taking it; needed a break"	14	3	5	2

7	"Didn't want friends asking questions; felt embarrassed"	13	6	4	1

8	"Nowhere to keep it at school or work"	15	4	4	1

9	"Not sure if dose was taken"	12	5	7	0

10	"Fell asleep"	16	4	4	0

11	"Got sick with another illness; wasn't feeling well"	20	1	3	0

12	"Ran out of medications"	19	1	4	0

## Results

Of the clinic's 50 staff members, 27 volunteered to participate in the simulated adherence exercise. The participating staff members included physicians (nine), nurses (eight), clinical support staff (eight), a pharmacist and a social worker. More than half (15) were from Swaziland, while the others were from North America (eight), Kenya (two), South Africa (one) and Germany (one). None had participated in an adherence exercise of this type before.

Adherence among participants was poor, with only one (4%) of the 27 participating Centre of Excellence staff achieving 95-100% overall adherence during the exercise. Though not possible to make a direct comparison, it is of interest that 46% of paediatric patients aged five years and younger and captured in the clinic's electronic medical record during the month of the exercise achieved 95-100% overall adherence.

On a five-level Likert scale ranging from "never" to "all of the time", 11 of the participants reported following his/her specific schedule "all of the time", while 13 reported following it "most of the time". The remaining three reported following the schedule "half" (one) or "some" (two) of the time. The top two reported barriers were being "too busy" and "family and/or friends don't help me remember".

The general response of the participants to the exercise was positive. Comments included: "This was an eye-opening exercise", "Very good for us to experience", and "Now I do relate with the challenges faced by our clients."

## Discussion

Informed adherence counselling is difficult without a first-hand appreciation of the difficulties inherent in administering regularly scheduled medications. While the clinic staff involved in this exercise understood the importance and complexity of adherence behaviours, few had personally experienced the challenges of daily adherence despite working directly with the end users of these medications on a daily basis.

The poor adherence rates among staff participants are likely a reflection of several factors. The primary objective of achieving good adherence among participants was not health related, and as a result, the incentives to achieve good adherence were not as strong as among parents of HIV-positive children. Also, the exercise lasted only a week, and so a single error in dosing has a potentially large effect on the calculated adherence. Moreover, most of the participants were handling paediatric ARVs for the first time.

The leading reported barriers among patients are similar to those previously reported. While careful scheduling and adherence support are routinely discussed at length in our pre-ART adherence counselling sessions, other challenges, such as difficulty drawing medicine and spillage, are often not addressed.

## Conclusions

Good adherence to liquid formulations of ARVs is challenging but essential in the treatment of paediatric HIV. When asked to adopt and simulate a typical paediatric ARV dosing schedule for a week-long exercise, our healthcare workers achieved poor adherence compared with our patient population, but identified with many of the barriers commonly reported by caregivers. With few opportunities to learn first hand what strict adherence to ARVs entails, simulations such as this have the potential to promote awareness of paediatric ARV adherence issues and empower healthcare staff to more effectively counsel caregivers and children taking ARVs, as well as other medications.

## Competing interests

The authors declare that they have no competing interests.

## Authors' contributions

BRP and SJH participated in the adherence exercise, data gathering, and initial manuscript development. JW revised the manuscript, which was finalized by GES. All authors read and approved the final manuscript.
